# Dysregulated Chaperone‐Mediated Autophagy and Impaired Autophagic Flux in Familial Alzheimer's Disease

**DOI:** 10.1002/alz70855_102522

**Published:** 2025-12-23

**Authors:** Maria‐del‐Carmen Cardenas‐Aguayo, Norma‐Adriana Lumbreras‐Zavala, Maria‐del‐Carmen Silva‐Lucero

**Affiliations:** ^1^ UNAM, School of Medicine, Department of Physiology, CDMX, DF, Mexico; ^2^ Posgrado de Neurobiología, UNAM, CMDX, DF, Mexico

## Abstract

**Background:**

The most prevalent cause of dementia is Alzheimer's disease (AD), which is histopathologically identified by intracellular neurofibrillary tangles made of the hyperphosphorylated tau protein and extracellular amyloid plaques made of the Amyloid‐β peptide (Aβ). Alzheimer's disease is divided into two categories: familial AD (FAD), also known as an early‐onset family disease (EOAD), which accounts for 1% of cases, and sporadic late‐onset (LOAD) disease, which accounts for over 99% of cases. Autosomal dominant mutations in the APP, PS1, and PS2 genes are the cause of FAD. Increased expression of macroautophagy markers and changes in signaling pathways linked to cellular stress, autophagy, lysosomes, and tau phosphorylation have been detected by our group and others in fibroblasts from FAD patients with a PS1 mutation (Lopez‐Toledo et al., 2022). Furthermore, autophagy malfunction and chaperone‐mediated autophagy (CMA) are linked to neurological diseases; for this reason, our study aims to assess macroautophagy and CMA in FAD fibroblasts.

**Method:**

The Coriell Institute (New Jersey) repository provided skin fibroblasts from FAD patients and non‐affected individuals, cultivated in Earl MEM salts medium containing 15% non‐inactivated FBS. Western blotting was used to characterize these cells' macroautophagy and CMA markers. Additionally, autophagy was initiated by fasting and chloroquine addition, and the Cyto‐ID essay and PremoTM Autophagy Tandem Sensor were used to measure the autophagic activity and autophagic flux, respectively.

**Result:**

Autophagy Assays indicated that FAD fibroblasts with very low autophagy flux (detected by the PremoTM Autophagy Tandem Sensor) had increased autophagy, as detected by an increased expression of LC3, as evidenced by variations in autophagy activity between cells from FAD patients and cells from healthy controls. Western blotting of CMA markers (LAMP2a and Hsc70) revealed that these markers were downregulated in FAD cells.

**Conclusion:**

When comparing the fibroblasts of FAD patients to those of apparently healthy individuals, autophagy activity and CMA markers revealed dysregulation. These peripheral cell discoveries may be helpful for drug testing, early diagnosis, and the discovery of novel biomarkers.

